# Kinematic and Joint Compliance Modeling Method to Improve Position Accuracy of a Robotic Vision System

**DOI:** 10.3390/s24082559

**Published:** 2024-04-16

**Authors:** Fan Ye, Guangpeng Jia, Yukun Wang, Xiaobo Chen, Juntong Xi

**Affiliations:** 1 School of Mechanical Engineering, Shanghai Jiao Tong University, Shanghai 200240, China; yefan019@sjtu.edu.cn (F.Y.); ykwangme@sjtu.edu.cn (Y.W.); xiaoboc@sjtu.edu.cn (X.C.); 2 China National Heavy Duty Truck Group Co., Ltd., No. 777 Hua’ao Road, Innovation Zone, Jinan 250101, China; jiaguangpeng@sinotruk.com

**Keywords:** robotic vision system, position accuracy, unified kinematic model, joint compliance model, error compensation, 3D laser sensor

## Abstract

In the field of robotic automation, achieving high position accuracy in robotic vision systems (RVSs) is a pivotal challenge that directly impacts the efficiency and effectiveness of industrial applications. This study introduces a comprehensive modeling approach that integrates kinematic and joint compliance factors to significantly enhance the position accuracy of a system. In the first place, we develop a unified kinematic model that effectively reduces the complexity and error accumulation associated with the calibration of robotic systems. At the heart of our approach is the formulation of a joint compliance model that meticulously accounts for the intricacies of the joint connector, the external load, and the self-weight of robotic links. By employing a novel 3D rotary laser sensor for precise error measurement and model calibration, our method offers a streamlined and efficient solution for the accurate integration of vision systems into robotic operations. The efficacy of our proposed models is validated through experiments conducted on a FANUC LR Mate 200iD robot, showcasing notable improvements in the position accuracy of robotic vision system. Our findings contribute a framework for the calibration and error compensation of RVS, holding significant potential for advancements in automated tasks requiring high precision.

## 1. Introduction

The advent of robotic vision systems (RVSs) has ushered in a new era of robotic capabilities, fundamentally transforming the scope and efficiency of automated tasks across various industries [1,2]. The intrinsic value of such systems lies in their ability to perform complex visual tasks with remarkable accuracy, from intricate assembly operations in manufacturing to delicate surgical procedures in medicine. High-precision RVSs enable robots to detect, recognize, and manipulate objects with a level of detail and accuracy previously unattainable, bridging the gap between robotic automation and tasks requiring human-like dexterity and visual acuity.

The position accuracy of an RVS is defined by the system’s capability to precisely locate a target within its field of view [3]. This accuracy is gauged by the system’s effectiveness in pinpointing an object’s position relative to its true location in the physical world. Enhancing this position accuracy necessitates robust modeling and calibration processes. These processes entail the acquisition of part point clouds via the vision system, followed by the transformation of these data points into a consistent representation of the target features within the Cartesian coordinate system. Therefore, meticulous modeling is paramount in improving the position accuracy of an RVS. Current methodologies for RVS modeling are bifurcated into kinematic and compliance models, each addressing different aspects of system behavior and contributing to the overall precision of the system [4].

The kinematic model primarily involves the identification of structural parameters of basic robotic movements [5] and the “hand eye” parameters [6,7] between the robot and camera coordinate systems. The D-H model proposed by Denavit and Hartenberg has become widely applied in kinematic modeling within the industrial robotics field, serving as a standard for an extended period [8,9]. Subsequent improvements to this model, such as those introduced by Hayati, incorporate angles between adjacent parallel joints [10]. Du Guanglong presents a novel approach for online robotic kinematic calibration, which, by integrating an Unscented Kalman Filter and an Iterative Particle Filter, achieves precise identification of robotic kinematic parameters without necessitating operational halts [11]. Joubair introduces a kinematic calibration approach utilizing distance and sphere constraints, significantly enhancing the position accuracy of a six-axis serial industrial robot within a specific target workspace [12]. The most common method for hand–eye calibration is based on estimating transformation matrices. Through reprojection error minimization, Koide directly utilizes calibration pattern images, without the need for explicit camera pose estimation [13]. Hua Jiang successfully demonstrates a robot hand–eye calibration approach utilizing an optimized neural network to accurately model the complex, nonlinear relationship between camera and robotic coordinates [14].

The robot compliance model focuses on the elasticity of the materials constituting the robot, addressing deformation errors under applied forces [15,16]. Due to its own weight and external loads, the robot experiences structural deformation in its links and joints [17]. Current research on the joint stiffness modeling of six-degree-of-freedom robots mainly analyzes deformations in joints 2 and 3, establishing simplified linear torsion spring models [18]. Abele utilized a linear torsion spring model and the Jacobian matrix to create a static compliance model for robots, enhancing tool path accuracy [19]. Dumas et al. developed a stiffness model for serial robots based on translational and rotational errors, experimentally validated on the Kuka KR240 robot, identifying compliance coefficients for its six joints [20]. P Kozlov used a CAD virtual experimental environment and finite element numerical analysis to derive the compliance matrices describing robot stiffness, achieving a comprehensive robot compliance model [21]. However, this model, which includes many redundant parameters, may not suit calibration involving numerous redundant parameters. Further, Klimchik et al. investigated the static calibration issue of heavily loaded industrial robots, employing the Virtual Joint Modeling method to establish a robot compliance model [22]. Du Liang developed an approach for calibrating compliance errors in robots by statistically analyzing the effects of gravity and elastostatic forces on individual joints. Utilizing single joint rotations and laser tracking measurements, the study identifies significant compliance errors and compensated for them, markedly improving robot accuracy and operational efficiency [23]. Tepper proposed a cost- and time-efficient approach for setting up a compliance model for industrial robots, utilizing an optimal design of experiments for variance-minimal Bayesian inference of gear stiffness parameters [24].

Overall, kinematic models aim to enhance RVS geometric accuracy with various solutions already available, while compliance models lack a unified consensus among researchers. There are four approaches to the compliance analysis of robots, incorporating Finite Element Analysis [25], the Virtual Joint Model [26], the nonlinear transmission model (NTM) [27], and the Rigid–Flexible Coupling Model (RFCM) [28]. Firstly, Finite Element Analysis simulates system parts to estimate deformation errors under different configurations but struggles with predicting errors from real shapes, material properties, and manufacturing assembly. Secondly, the Virtual Joint Model introduces excessive redundant parameters through adding 6-DOF springs at the joints, complicating identification. Thirdly, NTM corrects nonlinear errors of connectors in joint spaces, typically modeling end-effector spaces with high-order harmonic functions, which cannot solve the issues of interpretability and model under-fitting. Ultimately, RFCM considers the mechanical impact of robot self-weight and loads, but cannot indicate the mass and centroid errors of robot parts effectively.

As stated above, model calibration is indispensable for a newly installed or worn RVS. This study introduces a model-based kinematic and joint compliance approach, enhancing calibration and error compensation for precise positioning upon integrating vision systems into robots. Our contributions are as follows:

(1) We design a 3D rotary laser sensor mountable on robot grippers to create a representative RVS, propose an error measurement method based on vision measurement, and prove the ability to improve the position accuracy of the RVS.
(2) Existing RVS modeling methods separate the robot body and the hand–eye system calibration, leading to internal error accumulation. We proposed a unified kinematic model by trimming redundant structural parameters.
(3) We introduced a joint compliance model by combining NTM and RFCM, comprehensively considering the joint friction of connectors, external loads, and link self-weight on joint compliance. The specific optimization is as follows:
  (i) We proposed an extended NTM, using second-order Fourier functions to fit spatial errors of the terminal three joints based on the Pieper Criterion in robotics. And the under-fitting issue in NTM is addressed.
  (ii) To resolve the issue of model hyper-parameters caused by unknown external load configurations, we approximated the load’s position and direction by using the hand–eye transformation matrix of the RVS.
  (iii) Through mechanical analysis, we simplified the link model for self-weight.

To address these issues and propose kinematic and joint compliance modeling and calibration methods, this paper focuses on the establishment and parameter identification of RVS models. The rest of the paper is organized as follows: Section 2 introduces a 3D vision sensor to build the RVS, and establishes a unified kinematic model. Section 3 establishes the joint compliance model for the RVS, including an extended nonlinear transmission model and compliance models for the loads’ and links’ self-weights. Section 4 proposes a parameter identification process for kinematic and joint compliance models. Section 5 completes two accuracy verification experiments on the FANUC LR Mate 200iD robot. Section 6 concludes the paper.

## 2. Method Framework and Unified Kinematic Model

### 2.1. Overview of Kinematic and Joint Compliance Model

**Figure 1:**
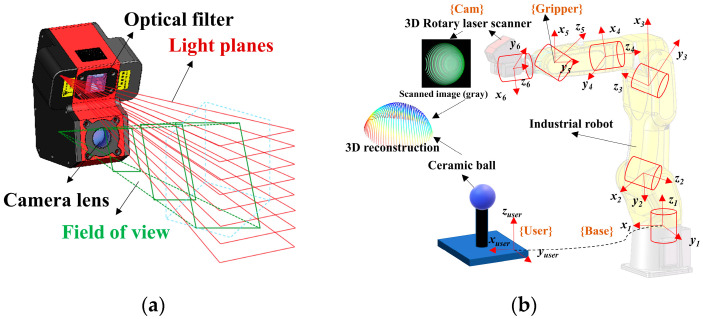
Overview of robotic vision system: (**a**) Design of the 3D rotary laser sensor. (**b**) Unified kinematic model.

**Figure 2:**
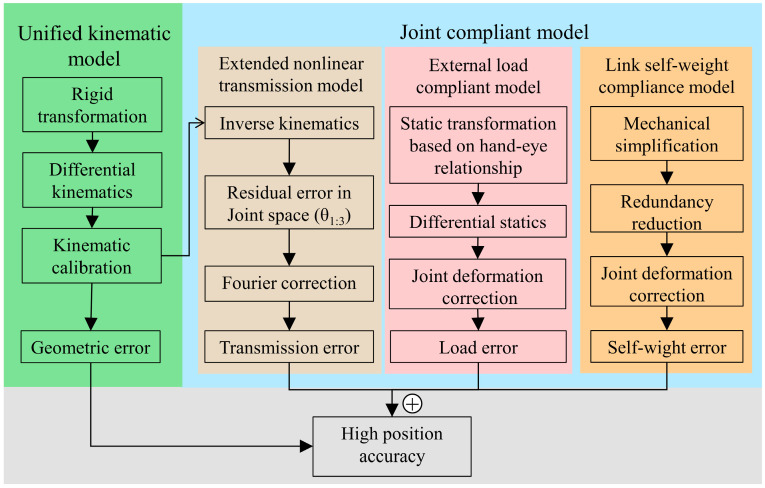
Modeling process of robotic vision system.

Figure 1 presents an overview of the robotic vision system in our study. A 3D rotary laser sensor is designed in Figure 1a, which is integrated into the gripper of a robot in Figure 1b. Moreover, Figure 2 provides a visual exposition of the methodological architecture underpinning the proposed kinematic and joint compliance model. The flowchart presents a structured sequence of operations beginning with a unified kinematic model that integrates both robot body modeling and hand–eye system calibration. This integration is critical to reducing internal error accumulation and serves as the basis for further error compensation strategies, ensuring a strong foundation for the precision of robotic movements and vision system coordination.

The subsequent step in the process involves the joint compliance model which addresses the elasticity of robot materials and corrects for deformation errors. This model accounts for joint connector elasticity, external load compliance, and the self-weight of robotic links, which are imperative for the robot’s operational accuracy under varying loads and conditions. The framework illustrates how these compliance factors are systematically incorporated into the model, providing a method to compensate for the various forces and torques affecting the robot’s joints. The symbols used in this paper are presented below.

### 2.2. Geometric Model of a 3D Rotary Laser Sensor

The calibration of industrial robot models requires reliable error observation methods. One mainstream approach is measuring the error of robot motion in configuration space (C-space) using 3D stereo vision sensors. Depending on the installation position of the vision sensor, there are two types: eye-in-hand and eye-to-hand. The choice between these two depends on the practical application requirements, with little difference in the underlying mathematical principles.

First, a geometric model of the rotary laser sensor is established. The camera imaging process includes both a distortion model and a geometric model. The geometric model of the camera is based on the pinhole imaging principle, mathematically expressed in Equation (1).

(1) $$\begin{array}{l} f_{x}X_{c}=\left(u-u_{0}\right)Z_{c}\\ f_{y}Y_{c}=\left(v-v_{0}\right)Z_{c} \end{array}$$

where $f_{x}$ and $f_{y}$ represent the camera’s focal lengths on the image plane, and $u_{0}$ and $v_{0}$ are the pixel coordinates of the intersection point between the camera’s optical axis and the image plane. $\left(u,v\right)$ and $\left(X_{c},Y_{c},Z_{c}\right)$, respectively, express the spatial point in the image plane coordinate system and the camera coordinate system. The distortion model can be expressed as Equation (2).

(2) $$\left\{\begin{array}{l} \overset{⌢}{u}=u\left(1+k_{1}r^{2}+k_{2}r^{4}+k_{3}r^{6}+2p_{1}v\right)+p_{2}\left(r^{2}+2u^{2}\right)\\ \overset{⌢}{v}=v\left(1+k_{1}r^{2}+k_{2}r^{4}+k_{3}r^{6}+2p_{2}u\right)+p_{1}\left(r^{2}+2v^{2}\right)\\ r=\sqrt{{\left(u-u_{0}\right)}^{2}+{\left(v-v_{0}\right)}^{2}} \end{array}\right.$$

where $\left(k_{1},k_{2},k_{3}\right)$ represents the radial distortion coefficient and the tangential distortion coefficient is $\left(p_{1},p_{2}\right)$. The radial distortion, influenced by Snell’s Law, addresses deviations from the optical axis, where light refracts to different positions, causing magnification discrepancies between actual and ideal imaging. And the tangential distortion coefficient compensates for errors caused by the nonparallel alignment of the lens and the imaging plane.

**Figure 3:**
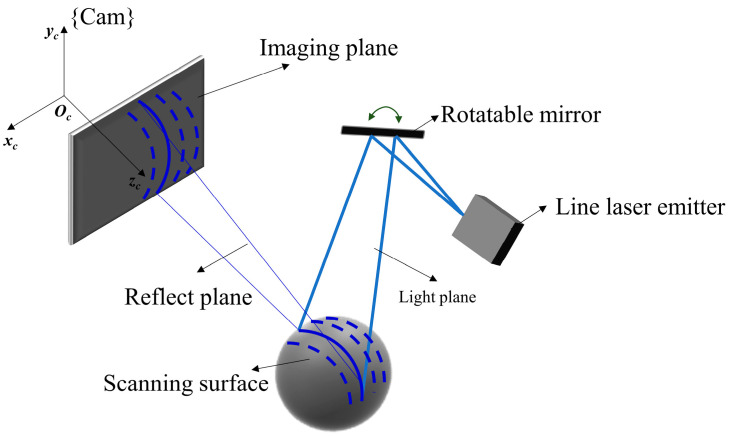
Schematic diagram of the 3D rotary laser sensor.

Subsequently, the structure of the 3D rotary laser sensor is designed in Figure 1a, and its schematic representation is illustrated in Figure 3. A line laser hits the mirror and the object surface in turn. Next, the light is captured by a fixed camera. Through the rotation of the mirror, multiple laser stripes can be illustrated in the image. The reflected light planes can be expressed as Equation (3).

(3) $${\vec{n}}^{i}{\left(\begin{array}{ccc} X_{c} & Y_{c} & Z_{c} \end{array}\right)}^{T}+{\left(\begin{array}{ccc} 0 & 0 & b^{i} \end{array}\right)}^{T}=0$$

where ${\vec{n}}^{i}$ is the normal plane to the light plane, and $b^{i}$ is the bias. Then, Equations (1) and (3) are expressed in matrix form, as shown in Equation (4).

(4) $$\left(\begin{array}{ccc} f_{x} & 0 & u_{0}-u\\ 0 & f_{y} & v_{0}-v\\ {n_{x}}^{i} & {n_{y}}^{i} & {n_{z}}^{i} \end{array}\right)\left(\begin{array}{c} X_{c}\\ Y_{c}\\ Z_{c} \end{array}\right)+\left(\begin{array}{c} 0\\ 0\\ b^{i} \end{array}\right)=0$$

Finally, the point cloud in camera frame {Cam} can be further calculated by 3D reconstruction Equation (5).

(5) $$\left(\begin{array}{c} X_{c}\\ Y_{c}\\ Z_{c} \end{array}\right)={\left(\begin{array}{ccc} f_{x} & 0 & u_{0}-u\\ 0 & f_{y} & v_{0}-v\\ {n_{x}}^{i} & {n_{y}}^{i} & {n_{z}}^{i} \end{array}\right)}^{-1}\left(\begin{array}{c} 0\\ 0\\ -b^{i} \end{array}\right)$$

### 2.3. Unified Kinematic Model

Methods of robot body modeling and hand–eye calibration have already been extensively studied individually. However, this fragmented approach introduces redundant kinematic parameters, leading to the accumulation of system errors.

This section builds upon the serial robot MDH (Modified Denavit–Hartenberg) model by introducing camera and user frames at the beginning and end of the robot, respectively. It further establishes a unified kinematic model for RVS that conforms to a physically expressive model with low redundancy. Then, a full parameter optimization solution is performed on the system’s kinematic model.

The initial model of the measurement system is illustrated in Figure 1b. The transformation from the camera frame {Cam} to the user frame {User} is defined as TCU, which satisfies Equation (6).

(6) q=TCUp

where p, q are the same targets described in {User} and {Cam}.

In order to separate the robotic model from the system, TCU can be divided into three parts. In Equation (7), TBU is the transformation matrix from the robot base frame {Base} to the {User}. TGB is the transformation matrix from the gripper frame {Gripper} to the {Base}. TCG is the transformation matrix from the {Cam} to the {Gripper}.

(7) TCU=TBUTGBTCG=T1U∏i=1JointsTi+1iTCJoints+1

where Joints is the number of robotic joints.

To reduce degrees of freedom, TBU and TCG are modeled based on the Euler angle, and TGB is constructed by the MDH model of the robot. The rotation and translation relationship is shown in Equations (8)–(10). In addition, parameters of the end joint are deleted because the 3D laser scanner is rigidly connected with the robot. Similarly, two redundant parameters near the robotic base are deleted, including $\theta_{1},d_{1}$.

(8) TBU=Transx0,y0,z0Rotzr0Rotyw0Rotxp0

(9) TCG=Transxe,ye,zeRotzreRotyweRotxpe

(10) TGB=Transa1,0,0Rotxα1∏i=2Joints−1RotzθiTransai,0,diRotxαi

where θ,d,a,α represent the joint angle, link offset, link distance, and twist angle, respectively. x,y,z,r,w,p denote three translations and three rotations of different directions. In summary, the established unified kinematic model includes 30 parameters $\left(\theta_{1}=d_{1}=0\right)$: $\eta_{\mathrm{Ki}}{=(x}_{0},y_{0},z_{0},r_{0},w_{0},p_{0}\cdots \theta_{i},d_{i},a_{i},\alpha_{i},\cdots x_{e},y_{e},z_{e},r_{e},w_{e},p_{e}{)}^{T},\left(i=1,2,\ldots ,5\right)$.

Based on $\eta_{\mathrm{Ki}}$, the kinematic coordinates of the robot’s position (defined as the origin of the frame {Cam}) can be obtained. Then, the unified kinematic model can be expressed as qKi=qηKi=TCU.

## 3. Joint Compliance Model

The unified kinematic model merely describes the geometric error of RVS, but is unable to cope with the elastic deformation. In general, global compliance models for various parts of the robot are established, based on elasticity theory used for nonrigid models. However, numerous redundant parameters will be introduced, causing under-fitting in calibration. According to [28], 90% of the elastic deformation in serial robotic arms concentrates at the joint connections. Thereby, a simplified form based on Hooke’s law of joint torsional elasticity is proposed in Equation (11).

(11) $$\delta \theta =\lambda_{\theta }\tau$$

where $\lambda_{\theta }$ is the compliant matrix of joint torsion, θ is a vector concatenated joint angle θ, and τ is a vector concatenated joint torque τ.

Analyzing the impact of each component from the perspective of joint compliance is of great significance. In quasi-static conditions, joint deformation is primarily influenced by three types of forces: frictional forces, externally applied loads, and the self-weight of the robot links. Based on the principle of superposition in mechanics, the effects originating from the torque output by each of the robot’s drive motors can be decomposed into joint frictional forces caused by connectors $\tau_{C}$, end-effector load forces $\tau_{F}$, and link gravitational forces $\tau_{\mathrm{Link}}$, as indicated in Equation (12).

(12) $$\tau =\sum_{i\in \mathrm{Joints}}\tau_{C}^{i}+\tau_{F}+\sum_{i\in \mathrm{Joints}}\tau_{\mathrm{Link}}^{i}$$

By combining (10) and (11), the joint compliance error can be derived as Equation (13).

(13) $$\delta \theta =\sum_{i\in \mathrm{Joints}}{\delta \theta }_{C}^{i}+{\delta \theta }_{F}+\sum_{i\in \mathrm{Joints}}{\delta \theta }_{\mathrm{Link}}^{i}$$

The joint frictional forces, originating from the relative rotation at joint connections, are influenced by several factors including motor encoders, gear reducers, and couplings. These forces are complex and challenging to explain through linear models. Consequently, this paper introduces a nonlinear transmission model to address the angle errors caused by frictional forces. Moreover, joint angle errors resulting from end-effector load forces and the gravitational forces of the robot links can be predicted using the theory of robot statics. Based on these predictions, a linear compliance model is established.

### 3.1. Extended Nonlinear Transmission Model

**Figure 4:**
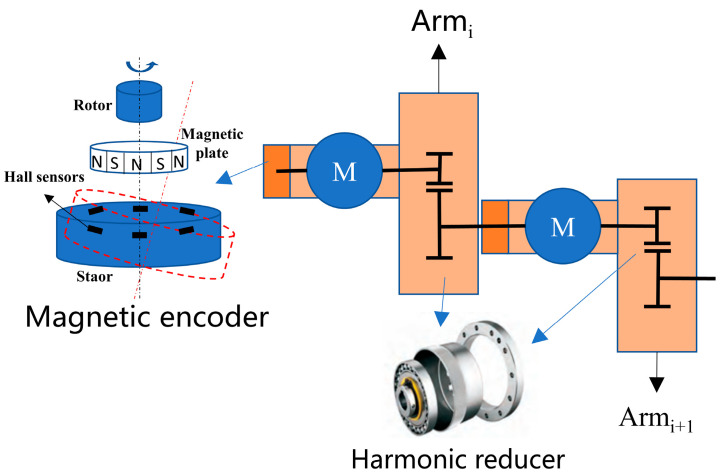
Basic composition of integrated joint modules.

An integrated joint module encompasses a motor, a magnetic encoder, and a harmonic reducer, where the encoder, attached to the motor’s shaft end, gauges the motor angle, and the motor shaft’s linkage to the harmonic reducer’s waveform generator facilitates output with reduced rotational speed through its flexible wheel. This setup introduces an error chain from the encoder through the harmonic reducer (as shown in Figure 4), affecting the joint module’s angular measurement precision due to both components. Manufacturing considerations for magnetic encoders include addressing the positioning inaccuracies in the magnetic plate’s pole distribution and the relative positioning of the Hall sensor and the magnetic plate, which are critical for the encoder’s signal accuracy. Imperfections such as tilt and eccentricity introduce periodic, low-frequency errors, necessitating harmonic analysis for error correction.

In addition, the harmonic reducer operates on elastic deformation principles, with a setup comprising rigid and flexible gears and a wave generator [27]. The wave generator, by driving the flexible gear and inducing deformation waves, achieves dynamic transmission through periodic elastic deformation, engaging with the fixed rigid gear. Transmission errors, including radial and run-out errors from the wave generator, geometric and motion eccentricity from gear machining and installation, and backlash between gears, manifest as periodic errors with distinct low- and high-frequency components. These characteristics justify employing a second-order Fourier analysis for detailed error assessment and mitigation in the manufacturing process [29].

Since the angular error of a joint module is caused by the elastic deformation of its components and has continuous and integrable characteristics over a cycle, the angular error of the joint module can be represented as a signal with a 2π period, approximated by the Fourier series $\varphi \left(\theta \right)$. As shown in Equation (14), the angular error of the connector $\delta \theta_{C}$, caused by the periodic change in angle, is transmitted to the robot’s tool center point (TCP) through the robot’s motion Jacobian matrix $J\left(\theta \right)$, and can ultimately be approximated as the positioning error $\delta q_{C}$ induced by frictional forces.

(14) $$\delta q_{C}=J\left(\theta \right)\delta \theta_{C}=J\left(\theta \right)\varphi \left(\theta \right)$$

In the equation, θ refers to the angle value measured by the robot’s magnetic encoder. Next, following the decomposition into a Fourier series, $\varphi \left(\theta \right)$ conforms to Equation (15).

(15) $$\varphi \left(\hat{\theta }\right)={\left(\varphi \left({\hat{\theta }}_{1}\right)\;\varphi \left({\hat{\theta }}_{2}\right)\;\cdots \;\varphi \left({\hat{\theta }}_{\mathrm{Joints}}\right)\right)}^{T}$$

where

(16) $$\varphi \left({\hat{\theta }}_{j}\right)=k_{0}^{\left(j\right)}+\sum_{i=1}^{N}k_{\mathrm{ai}}^{\left(j\right)}\cos\left(iw{\hat{\theta }}_{j}\right)+\sum_{i=1}^{N}k_{\mathrm{bi}}^{\left(j\right)}\sin\left(iw{\hat{\theta }}_{j}\right)$$

In the equation, N represents the order of the Fourier series, k is the amplitude of the i-th order component signal, and j denotes the joint index. For simplicity, w will be forced to 1.

To solve for k in Equation (16), taking a six-degree-of-freedom industrial robot as an example, it is necessary to calculate the error $\varphi \left({\hat{\theta }}_{j}\right)$ for each joint of the robot through inverse kinematics. Since inverse kinematics involves mapping from Cartesian space to joint space, to ensure the inverse kinematics solving process adheres to ${\mathbb{R}}^{3}\to {\mathbb{R}}^{3}$, this paper restricts the three degrees of freedom in the joint space based on the Pieper Criterion (the axes of the last three joints of a six-degree-of-freedom robot always intersect at a single point, which has a minor impact on positional errors.), denoted as $\varphi \left({\hat{\theta }}_{4}\right)=\varphi \left({\hat{\theta }}_{5}\right)=\varphi \left({\hat{\theta }}_{6}\right)=0,\;N=2$.

After simplification, $\varphi \left(\theta \right)$ can be expressed as Equation (17). And the parameters of the extended NTM are expressed as $\eta_{C}=\left(k_{0}^{\left(1:3\right)},k_{a1}^{\left(1:3\right)},k_{b1}^{\left(1:3\right)},k_{a2}^{\left(1:3\right)},k_{b2}^{\left(1:3\right)}\right)$.

(17) $$\begin{array}{l} \varphi \left({\hat{\theta }}_{1:3}\right)=k_{0}^{\left(1:3\right)}+k_{a1}^{\left(1:3\right)}\cos\left(\hat{\theta }\right)+k_{b1}^{\left(1:3\right)}\sin\left(\hat{\theta }\right)\\ \;\;\;\;\;\;+k_{a2}^{\left(1:3\right)}\cos\left(2\hat{\theta }\right)+k_{b2}^{\left(1:3\right)}\sin\left(2\hat{\theta }\right) \end{array}$$

### 3.2. Compliance Error Caused by the External Load

**Figure 5:**
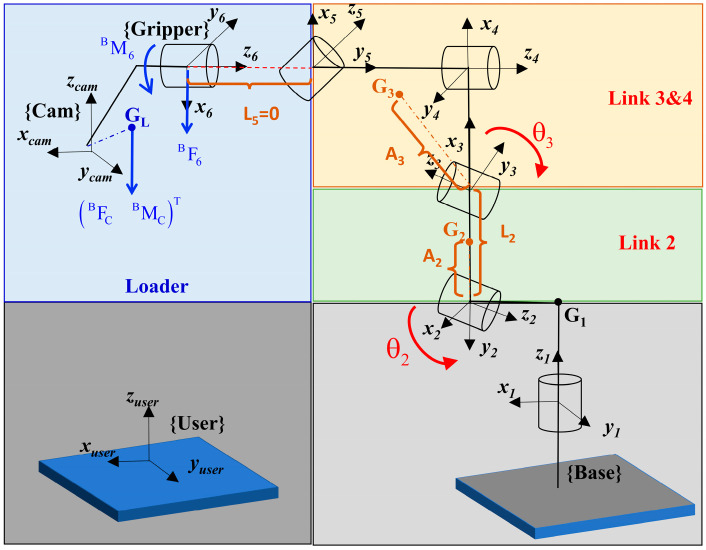
Mechanical effects of external load and robot links.

After modeling the nonlinearities of the transmission system, this section focuses on modeling the linear part of joint deformation. Based on the theory of linear torsional springs, a fixed stiffness coefficient is assigned to each joint of the robot. To establish the mathematical relationship between the forces and deformations experienced by the robot’s end effector in various configurations of the robot and the external load, it is first necessary to understand the forces and deformations at each of the robot’s joints. Figure 5 shows the mechanical relationship.

In the robot’s base frame {Base}, assume that the force applied at the origin of the camera frame {Cam} is FCB  MCBT, and the force applied at the origin of the end actuated joint frame {Gripper} is FGB  MGBT. For the six-degree-of-freedom (6-DOF) serial industrial robot in this paper, we set G = 6. The relationship between them can be solved by force-and-torque transformation HBC6, as shown in Equation (18).

(18) F6BM6B=HBC6FCBMCB

The transformation of forces under frame conversion HBC6 is seen in Equation (19).

(19) HBC6=E30tC6RC6+ΔC6E3

where tC6 and RC6 are the translation and rotation matrices of TCG in Equation (9); specifically, tC6=xe,ye,zeT and RC6=RotzreRotyweRotxpe. ΔC6 is the error matrix from the load center of gravity to the origin of {Cam}. We assume that ΔC6 is equal to zero, because it mainly affects the torque of the sixth joint with a relatively short lever arm, and has minimal impact on the other joints.

The notation $\left[t\right]$ in Equation (19) represents the antisymmetric matrix of the three-dimensional vector t, and when $t={\left(\begin{array}{ccc} t_{x} & t_{y} & t_{z} \end{array}\right)}^{T}$, [t] can be expressed using Equation (20).

(20) $$\left[t\right]=\left(\begin{array}{ccc} 0 & -t_{z} & t_{y}\\ t_{z} & 0 & -t_{x}\\ -t_{y} & t_{x} & 0 \end{array}\right)$$

For the high-precision positioning requirements of RVS in noncontact scenarios, the gravity of the externally applied load acts in a vertically downward direction. The force acting on the center of gravity of the externally applied load can be described in the frame {Cam} as FCBMCBT=00−mLg000T.

Based on the definition of the robot kinematics Jacobian matrix $J\left(\theta \right)$, and the relationship between it and the static-force Jacobian matrix $J_{F}\left(\theta \right)$, the errors caused by the load $\delta q_{F}$ and the load torque $\tau_{F}$ can be derived, as shown in Equation (21).

(21) δqF=JθδθFτF=JTθF6BM6B=JFθF6BM6B

Based on the linear torsion spring theory, the compliance coefficient corresponding to each joint is denoted as $\lambda_{\theta }$. According to the linear torque–torsion relationship, the relationship between the torsional torque of each joint and its elastic deformation can be obtained as shown in Equation (22).

(22) $$\delta \theta =\lambda_{\theta }\tau =\left(\begin{array}{ccc} \lambda_{1} & & \\ & \ddots & \\ & & \lambda_{\mathrm{Joints}} \end{array}\right)\tau$$

where $\delta \theta ={\left(\begin{array}{cccc} \delta \theta_{1} & \delta \theta_{2} & \cdots & \delta \theta_{\mathrm{Joints}} \end{array}\right)}^{T}$ represents the elastic deformation of each joint under torsional torque, $\lambda_{\theta }$ denotes the compliance coefficient of each joint, and $\tau ={\left(\begin{array}{cccc} \tau_{1} & \tau_{2} & \cdots & \tau_{\mathrm{Joint}} \end{array}\right)}^{T}$ is the torsional torque corresponding to each joint.

By combining Equations (16), (19), and (21), the pose error under the base frame {Base} of the 6-DOF industrial robot studied in this paper can be expressed as Equation (23).

(23) δqF=JθλθJTθHBC6FCB0

For an RVS without external forces, the direction of the load aligns with the direction of gravity; that is, FCBFCB=00−1T=−e3. By integrating the load weight FCB with the compliance matrix $\lambda_{\theta }$ to form λθ∗=FCBλθ, Equation (24) can be derived. The parameters of the external load compliance model are expressed as $\eta_{F}=\left(\lambda_{1}^{*},\lambda_{2}^{*},\lambda_{3}^{*},\lambda_{4}^{*},\lambda_{5}^{*},\lambda_{6}^{*}\right)$.

(24) δqF=JθFCBλθJTθHBC6FCBFCB0=Jθλθ∗JTθHBC6−e30

### 3.3. Compliance Error Caused by Weight of Robot Link

Furthermore, the effect of the self-weight of the links on joint deformation is crucial. As the robot is in any position or posture, the joint torque generated by the self-weight of the links always exists, and the compliance error caused by this factor is always coupled with geometric parameter errors. The body structure of most industrial serial robots is similar to that of the FANUC LR Mate 200iD robot, allowing for a generalized analysis method. The centroid of the link relative to the origin of the robot’s base frame {Base} forms a cantilever structure, generating torque. For a particular joint, its compliance error accumulates unidirectionally, for example, joint 3 is affected by the self-weight of links 3, 4, 5, and 6. The torque that does not change with the robot’s posture can be represented by the kinematic model. Therefore, two scenarios can be disregarded: (1) the lever arm between the link and gravity is zero; (2) the rate of change in the lever arm between the link and gravity is zero.

As shown in Figure 5, the rotation center axis (z_1_) of link 1 is parallel to the gravity vector, so the self-weight’s torque effect on the joint can be ignored. The rotation center axes (z_3_, z_4_) of links 2 and 3 are approximately perpendicular to the gravity vector, and their self-weight effects cannot be ignored. When link 4 rotates around its rotation center axis (z_4_), the lever arm does not change, so its effect on joint 4 can be ignored. The frame origins of links 5 and 6 coincide (L_5_ = 0 mm), and link 6 is rigidly connected to the load, so the self-weight effects of links 5 and 6 are already included in Equation (22). Therefore, only the deformations produced by the self-weight of links 2, 3, and 4 on joints 2 and 3 need to be considered.

Define the distance from the centroid G_2_ of link _2_ to its rotation center axis (z_2_) as A_2_, and the distance from the centroid to the rotation center axis (z_3_) of link _3_ as A_3_. Consider links 3 and 4 as a whole, with their corresponding centroid as G_3_. The lever arms exerted by each link on the joint are shown in Table 1.

By summing the torque components experienced by each joint as listed in Table 1, Equation (25) can be derived in matrix form.

(25) $$\begin{array}{l} \tau_{\mathrm{Link}23}=\left(\begin{array}{ccc} \sin\left(\theta_{2}\right) & \sin\left(\theta_{2}-\theta_{3}\right) & 0\\ 0 & 0 & \sin\left(\theta_{2}-\theta_{3}\right) \end{array}\right)\left(\begin{array}{c} G_{2}A_{2}+G_{3}L_{2}\\ G_{3}A_{3}\\ G_{3}A_{3} \end{array}\right)\\ \;\;\;=N_{23}\left(\theta \right)\left(\begin{array}{c} s_{1}\\ s_{2}\\ s_{2} \end{array}\right) \end{array}$$

By substituting Equation (21) into Equation (25), the end-effector position error caused by the self-weight of the links can be obtained as Equation (26).

(26) $$\begin{array}{ll} \delta q_{\mathrm{Link}23} & =J_{23}\left(\theta \right)\left(\begin{array}{cc} \lambda_{2} & 0\\ 0 & \lambda_{3} \end{array}\right)N_{23}\left(\theta \right)\left(\begin{array}{c} s_{1}\\ s_{2}\\ s_{2} \end{array}\right)\\ & =J_{23}\left(\theta \right)\lambda_{23}^{*}N_{23}\left(\theta \right)\left(\begin{array}{c} {s_{1}}^{*}\\ {s_{2}}^{*}\\ {s_{2}}^{*} \end{array}\right) \end{array}$$

where s1∗=s1FCB−1, s2∗=s2FCB−1, and λ23∗=FCBdiagλ2,λ3. And the parameters of the robot link self-weight compliance model are expressed as $\eta_{\mathrm{Link}}=\left(s_{1}^{*},s_{2}^{*}\right)$.

In summary, combining Equations (7), (17), (24), and (26), the kinematic and joint compliance model can be expressed as Equation (27) in differential form.

(27) $$\delta q=\delta q_{Ki}+\delta q_{C}+\delta q_{F}+\delta q_{\mathrm{Link}23}$$

## 4. Parameter Identification

**Figure 6:**
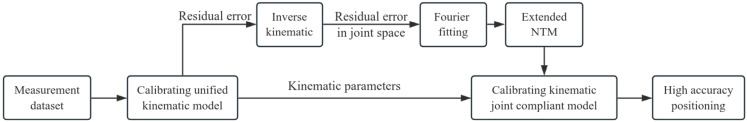
Process of parameter identification.

The models proposed in Section 2 and Section 3 establish the mathematical relationship between the parameters to be identified and the RVS across different poses. However, it is difficult to identify all parameters simultaneously, especially since the extended NTM in Section 3.1 has high nonlinearity. Therefore, parameter identification is required in multiple stages in Figure 6.

The process begins with a measurement dataset to calibrate the unified kinematic model as the foundation for identifying the parameters of the joint compliance model. This step is crucial as it establishes the kinematic parameters that are the bedrock for subsequent procedures. Next, the process splits into two branches. In the first branch, the inverse kinematics are computed, which leads to the determination of the residual error of joint angles. This error is then addressed through Fourier fitting, which helps to fine-tune the extended nonlinear transmission model (NTM). In parallel, the second branch is focused on calibrating the whole kinematic and joint compliance model, which integrates the kinematics of the robot with the mechanical effects of transmission, load, and the robot’s own links. Finally, an accurate model is obtained, which represents a synergy of all the calibrated parameters and models.

The parameter Jacobian matrix $J\left(\eta \right)$ is necessary for identification, based on the differential kinematics model. For a single measurement point, it includes three scalar equations, which can be represented as Equation (28).

(28) $$\left(\begin{array}{c} \Delta q_{1}\\ \Delta q_{2}\\ \vdots \\ \Delta q_{n} \end{array}\right)=\left(\begin{array}{cccc} \frac{\partial q_{1}}{\partial \eta_{1}} & \frac{\partial q_{1}}{\partial \eta_{2}} & \cdots & \frac{\partial q_{1}}{\partial \eta_{m}}\\ \frac{\partial q_{2}}{\partial \eta_{1}} & \frac{\partial q_{2}}{\partial \eta_{2}} & \cdots & \frac{\partial q_{2}}{\partial \eta_{m}}\\ \vdots & \vdots & \vdots & \vdots \\ \frac{\partial q_{n}}{\partial \eta_{1}} & \frac{\partial q_{n}}{\partial \eta_{2}} & \cdots & \frac{\partial q_{n}}{\partial \eta_{m}} \end{array}\right)\left(\begin{array}{c} \Delta \eta_{1}\\ {\Delta \eta }_{2}\\ \vdots \\ {\Delta \eta }_{m} \end{array}\right)=J\left(\eta \right)\Delta \eta$$

where Δq is the position error that can be physically measured in the frame {User}, and it can be substituted by the measurement error Δp in the frame {Cam}. Δη is the errors associated with the model parameters.

The identification of η is a nonlinear estimation problem, which can be calculated by the Levenberg–Marquardt (L-M) algorithm. An iterative gradient descent algorithm is summarized as follows:

(1) Calculate the parameter Jacobian matrix $J\left(\eta \right)$.
(2) Calculate the update vector $\Delta \eta_{k}$ of parameter:
  $$\Delta \eta_{k}={\left(J^{T}\left(\eta_{k}\right)J\left(\eta_{k}\right)\right)}^{-1}J^{T}\left(\eta_{k}\right)\Delta q$$
(3) Update: $\Delta \eta_{k+1}=\eta_{k}+\zeta \Delta \eta_{k},k=k+1$

where ζ is the descent rate of each iteration and equals 0.005.

According to the steps in Figure 6, combined with Equation (28) and the L-M algorithm, the parameter identification is performed in three stages:

Stage 1: Calibrate the unified kinematic model and identify 30 unified kinematic parameters $\eta_{K}$. After the calibration, the residual errors of each measurement point are recorded as $\left(\Delta r_{x},\Delta r_{y},\Delta r_{z}\right)$.

Stage 2: Calibrate the extended NTM. First, the residual errors of the unified kinematic model are projected into the joint space, based on the numerical inverse kinematics in Equation (29). Furthermore, the parameters of the extended NTM $\eta_{C}$ are identified by discrete Fourier transform.

(29) $$\left(\begin{array}{c} \Delta r_{x}\\ \Delta r_{y}\\ \Delta r_{z} \end{array}\right)=\left(\begin{array}{ccc} \frac{\partial q_{x}}{\partial \theta_{1}} & \frac{\partial q_{x}}{\partial \theta_{2}} & \frac{\partial q_{x}}{\partial \theta_{3}}\\ \frac{\partial q_{y}}{\partial \theta_{1}} & \frac{\partial q_{y}}{\partial \theta_{2}} & \frac{\partial q_{y}}{\partial \theta_{3}}\\ \frac{\partial q_{z}}{\partial \theta_{1}} & \frac{\partial q_{z}}{\partial \theta_{2}} & \frac{\partial q_{z}}{\partial \theta_{3}} \end{array}\right)\left(\begin{array}{c} {\Delta \theta }_{1}\\ {\Delta \theta }_{2}\\ {\Delta \theta }_{3} \end{array}\right)=J^{†}\left(\theta \right)\Delta \theta$$

where $\left(\Delta \theta_{1},\Delta \theta_{2},\Delta \theta_{3}\right)$ are the angular residual errors of the point in the joint space (J1–J3). $J^{†}\left(\theta \right)$ is the numerical Jacobian matrix of the joint angles.

Stage 3: All 53 parameters of the kinematic and joint compliance model $\eta ={\left(\eta_{\mathrm{Ki}},\eta_{C},\eta_{F},\eta_{\mathrm{Link}}\right)}^{T}$ are identified to obtain the optimal position accuracy.

## 5. Calibration Experiment of Kinematic and Joint Compliance Model

**Figure 7:**
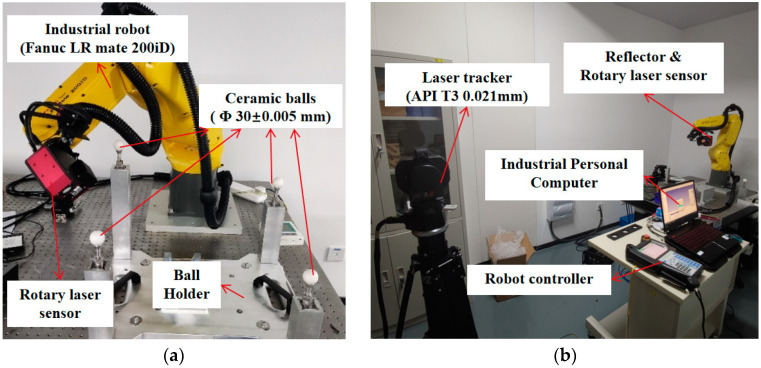
Experiment setups: (**a**) calibration platform based on the 3D rotary laser sensor; (**b**) calibration platform based on a laser tracker.

To assess the improvement in system position accuracy provided by our proposed method, we first calculated the geometric parameters of the 3D rotary laser sensor based on Zhang’s calibration method, and completed the local accuracy measurement verification of the four ceramic sphere calibrators. Then, we set up two experiments to validate the position accuracy of our model, as shown in Figure 7.

The first experiment was conducted with a calibrator composed of four ceramic balls, in order to assess the overall performance of the RVS. These four balls were measured by a Coordinate Measuring Machine (Hexagon Leitz PMM-Xi) with a precision of 0.5 μm, as shown in Table 2. The robot, carrying the rotating laser sensor, scanned the point cloud of the standard ceramic balls from different poses and calculated the center of the spheres. And a set of data points under the camera frame {Cam} were produced, which correspond to the four data points under the ceramic ball frame {Ceramic}.

The second experiment was conducted with a laser tracker to eliminate the influence of the 3D rotary laser sensor. We mounted the reflector of the laser tracker on the side panel of the sensor. Then, the robot was controlled to move to 7000 array positions. The array points under the laser tracker’s frame {LT} ere obtained, which correspond to the origin under the reflector’s frame {Reflector}.

### 5.1. Calibration of 3D Rotary Laser Sensor

In our previous work [30], the principle and feasibility of geometric feature measurement using the 3D rotary laser sensor were already validated and will not be elaborated upon here. Therefore, this section will focus on verifying the accuracy of the sensor in measuring ceramic spheres.

In the experimental setup, the 3D rotary laser sensor employed comprised an industrial camera with model number MV-CA013-20GM, offering a resolution of 1280 × 1024 pixels. The camera was capable of capturing images at a frame rate of 90 Hz, with a 16mm focal length providing a horizontal field of view (HFOV) of 21.7° and a vertical field of view (VFOV) of 17.5°. The sensor’s laser component had an output power of 50 mW at a wavelength of 405 nm. A rotating mirror, integral to the sensor, rotated at an angle of 20°, facilitating the sensor’s ability to create 120 distinct flight planes for data acquisition. The sensor’s optimal working distance was set between 115 and 135 mm, with a baseline distance of 69 mm. The system offered high resolutions, with a horizontal resolution of 0.5 mm and an even finer vertical resolution of 0.045 mm, ensuring detailed and precise data collection for the study.

**Figure 8:**
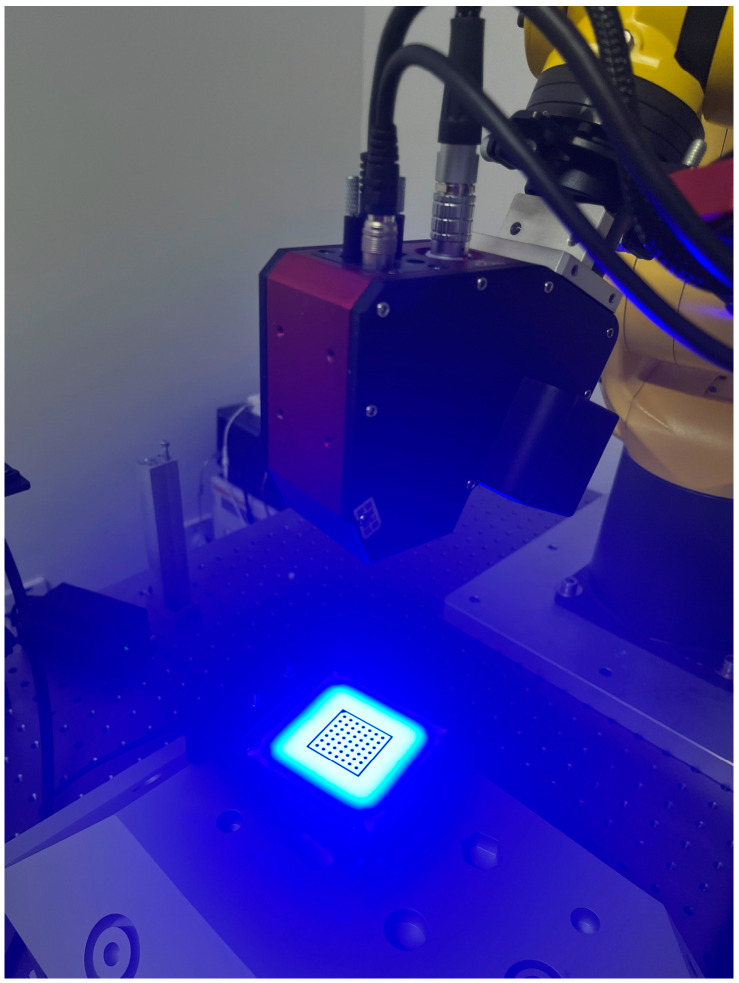
Geometric parameter calibration of the 3D rotary laser sensor.

As shown in Figure 8, a backlight chessboard was used to calibrate the 3D rotary sensor based on Zhang’s algorithm. The intrinsic parameters of the camera utilized in the 3D rotary laser sensor were meticulously calibrated to ensure the accuracy of the experimental data. The focal lengths in the x and y axes were determined to be $f_{x}$ = 3423.018 and $f_{y}$ = 3420.949, respectively. The principal point of the camera, which is the point on the sensor where the optical axis intersects, was located at coordinates $\left(u_{0},v_{0}\right)$ = 605.260, 580.506. The radial distortion coefficients $\left(k_{1},k_{2},k_{3}\right)$, critical for correcting lens distortions, were found to be −0.163, 0.951, and −0.874. Additionally, the tangential distortion coefficients $\left(p_{1},p_{2}\right)$ were measured as 0.000107 and −0.00119, which are imperative for correcting the decentering distortion in the camera lens system. According to the calibration result of 3D rotary sensor, the re-projection error of the camera is 0.06 pixels, and the average fitting error of 120 light planes is 0.004 mm.

**Figure 9:**
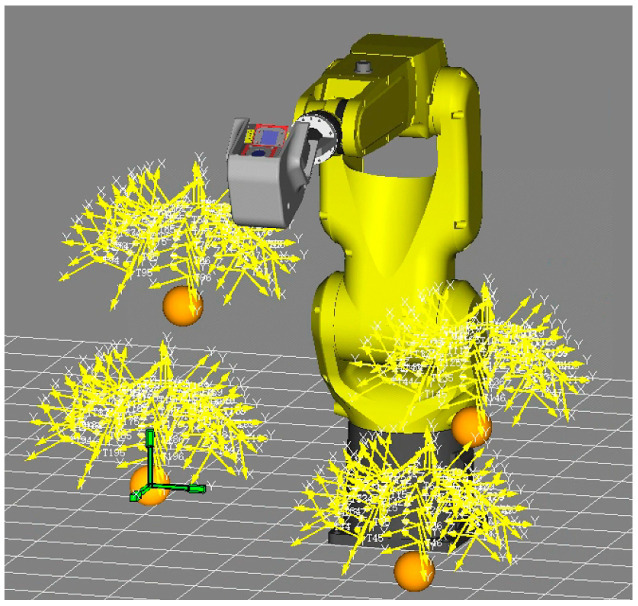
Measurement poses around ceramic balls.

**Figure 10:**
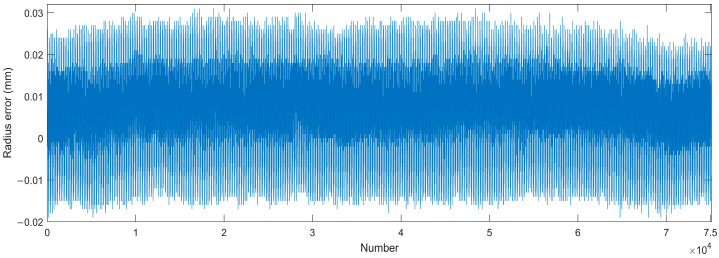
The radius error of the measured ceramic balls (4 balls × 50 poses × 372 rounds).

Then, the robot moved the 3D rotary sensor to measure 200 positions around the upper part of the four ceramic balls from different directions, following the layout method in [12]. Additionally, all positions were simulated in WeldPro and distributed as evenly as possible in Figure 9. According to 372 rounds of cycle measurements of the surface, the position of the balls’ center can be optimized using the Spherical Regression Algorithm. Next, the radius error distribution is concluded in Figure 10. The RMS (Root Mean Square) of the shape accuracy is 0.0114 mm and the Std. (standard deviation) can reach 0.0049 mm.

### 5.2. Accuracy Verification Based on 3D Rotary Laser Sensor

From the first experiment shown in Figure 7a, we validated the position accuracy of the RVS by measuring the four ceramic spheres with the 3D rotary laser sensor in Figure 8. According to the 200 measured points of ceramic spheres from different directions, 100 random positions were used for system calibration, and the remaining 100 for evaluation.

After the parameter identification process in Section 4, the kinematic and joint compliant model calibration results of RVS were calculated, including two frame transformations listed in Table 3, and the MDH parameters and joint compliance parameters in Table 4.

**Figure 11:**
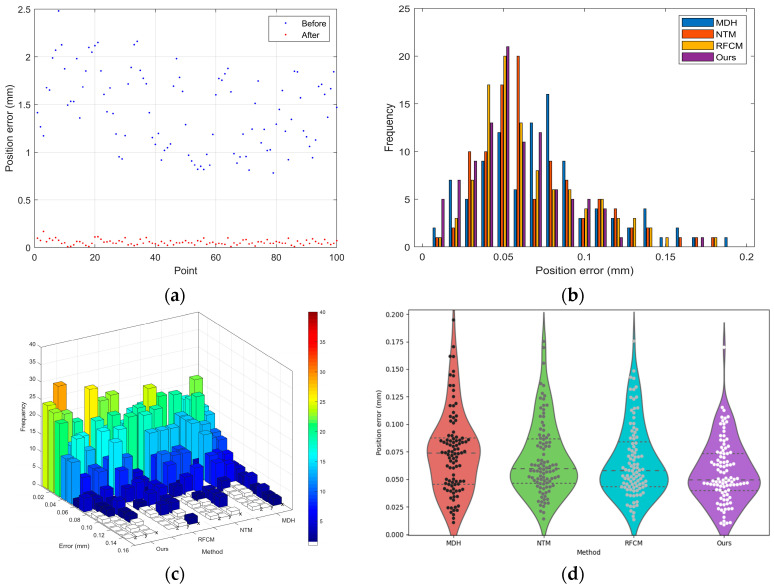
Results of first experiment: (**a**) position errors before and after calibration; (**b**) count of position errors; (**c**) count of errors in different directions; (**d**) error distribution.

The results depicted in Figure 11, alongside the data presented in Table 5, provide a quantitative and visual assessment of our calibration method’s performance. Figure 11a shows a scatter plot of position errors before and after calibration at different points, with a noticeable reduction in error after calibration, signifying an improvement in accuracy post calibration. Figure 11b illustrates a histogram of the frequency of position errors, comparing our method with three others: MDH [31], NTM [27], and RFCM [17]. Our method demonstrates fewer occurrences of higher errors, with the majority of errors congregating towards the lower end of the scale. Figure 11c is a 3D bar chart, offering a visual comparison of error frequencies for various calibration methods at different directions. Our method consistently shows lower frequency counts for larger errors, further emphasizing its precision. Figure 11d is a violin plot providing a visual summary of error distribution for each method. It reveals that our method has a tighter distribution of errors, suggesting a higher level of precision and reliability. The majority of the data points are clustered near the lower end of the error scale, and the spread of data points is narrower, indicating fewer outliers and less variation in measurement error.

Table 5 shows that our method has achieved a reduction in the Root Mean Square (RMS) error, outperforming other methods. Specifically, our method exhibits a 25.7% RMS improvement over the MDH method and surpasses the NTM and RFCM methods by 18.2% and 17.1%, respectively. When measuring with the 3D rotary laser sensor, the reductions in standard deviation and the average and maximum errors corroborate the superior calibration performance of our method, suggesting that it is not only more accurate on average but also more consistent and reliable across a range of measurements.

### 5.3. Accuracy Verification Based on a Laser Tracker

To eliminate the influence of measurement errors from the 3D rotary laser sensor, we used a laser tracker as the accuracy verification device, for which we designed the measurement scheme shown in Figure 7b. We affixed the ball seat of the laser tracker onto the 3D rotary laser sensor to measure positions but not attitude data. Subsequently, the joint angles from the FANUC robot register and the positions of the reflector were collected.

**Figure 12:**
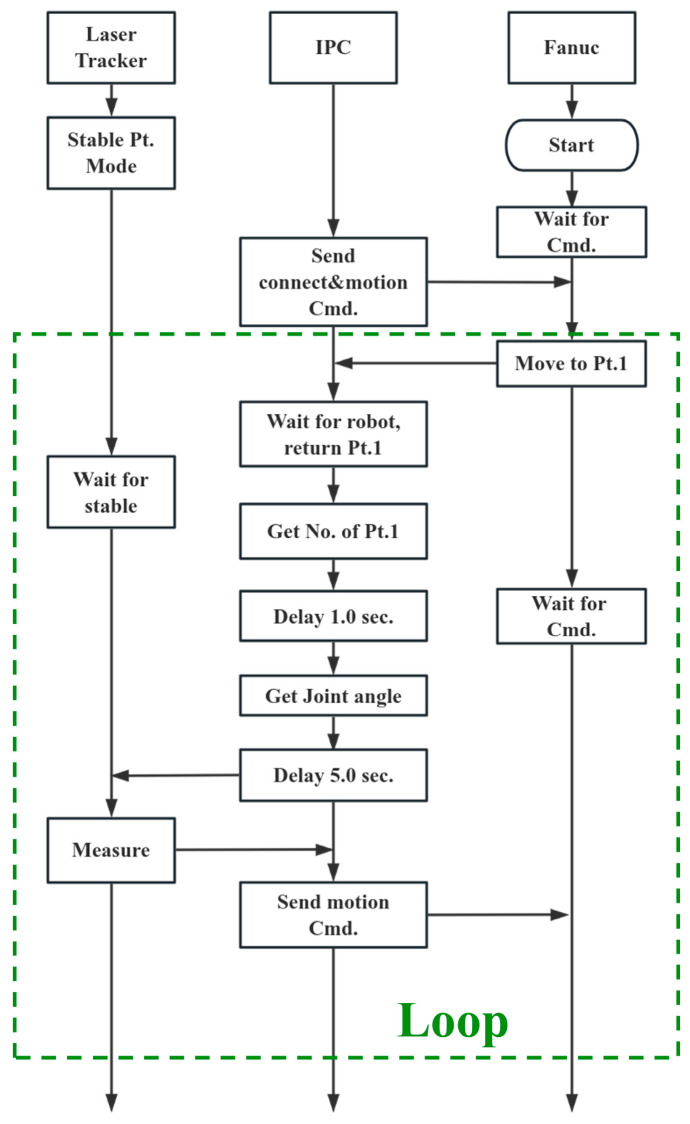
Process of automated measurement.

The automated measurement process is illustrated in Figure 12. We utilized an Industrial PC (IPC) to control and collect path data from the FANUC robot and the laser tracker (API T3). Communication with the robot’s string registers via the IPC allows for the control of the robot to execute pre-defined simulation paths and read the six angles from the robot joint encoders. When the robot moves to position Pt., it sends a signal to the IPC. A 6.0 s data-reading pause is reserved to ensure the robot stabilizes. The laser tracker is set to Stable Point Mode, automatically locating the reflector mounted on the 3D rotary laser sensor when the robot stops steadily. The advantage of this scheme is that it allows for direct evaluation of the kinematic and joint compliance model without introducing measurement errors from the 3D rotary laser sensor. Meanwhile, due to the single-point tracking lacking the reflector’s attitude, it is necessary to modify the kinematic model: the $r_{e},w_{e},p_{e}$ of TCG in Equation (9) is set to 0.

**Figure 13:**
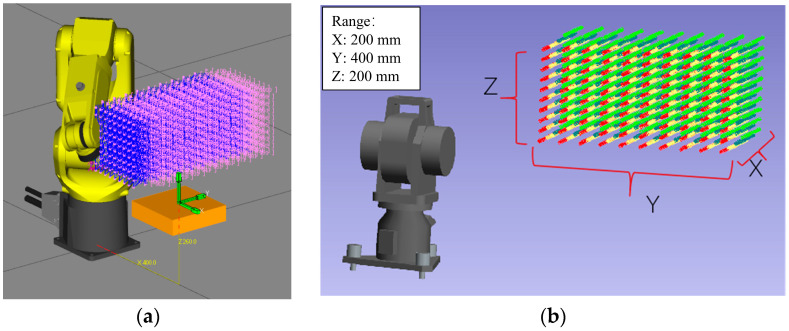
Measurement points: (**a**) path planning for robots; (**b**) results in laser trackers (the color correspond to the orientation of robot).

**Figure 14:**
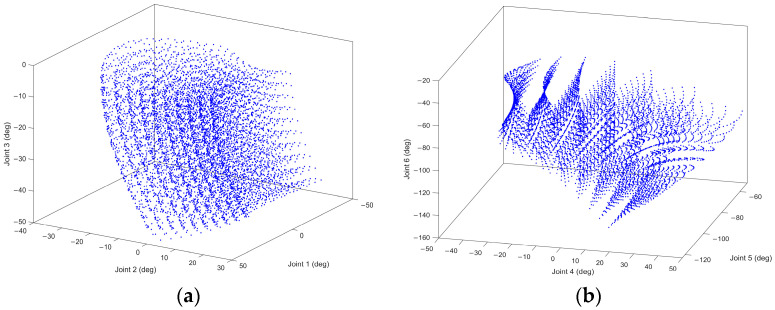
Distribution of the measurement points in the joint space: (**a**) Joint 1–Joint 2–Joint 3; (**b**) Joint 4–Joint 5–Joint 6.

Then, the laser tracker is used to measure 7000 points in a spatial array as reference data, as shown in Figure 13. Following the layout design in references [32,33], these 7000 points are arranged in seven robot orientations, with each pose set up in a 10³ configuration. The final distribution of the measurement points across the six joint spaces of the robot is depicted in Figure 14, with the least active joint reaching an approximate range of 50°.

As a result, 6998 effective position points were measured, of which 100 random points were selected for calibrating our model. Following the calibration procedure described in Section 4, TBU and TCG in the unified kinematic model are identified in Table 6. Meanwhile, the identification results of the MDH and joint compliant parameters are presented in Table 7.

Comparing the results of the two experiments in Table 4 and Table 7, it can be observed that there is a significant difference in the 15 parameters $k^{\left(1:3\right)}$ related to the extended NTM, while the differences in $\lambda_{\theta }^{*},s_{1}^{*},s_{2}^{*}$ are smaller. It is speculated that the reason for this is the smaller data volume in the second experiment, leading to larger errors in $k^{\left(1:3\right)}$ due to Fourier analysis, and part of the transmission model error being transmitted to $\lambda_{\theta }^{*},s_{1}^{*},s_{2}^{*}$.

**Figure 15:**
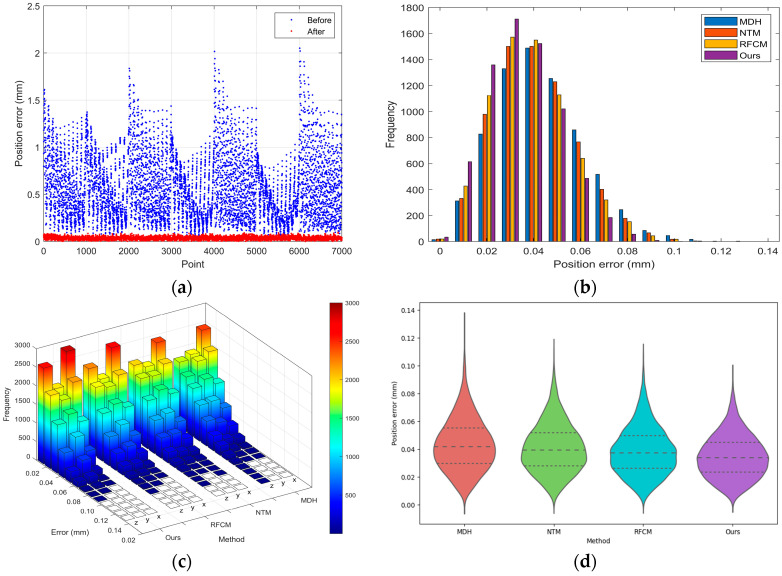
Results of second experiment: (**a**) position errors before and after calibration; (**b**) count of position errors; (**c**) count of errors in different directions; (**d**) error distribution.

The accuracy of the kinematic and joint compliance modeling method is evaluated based on the 6998 points. The experimental results are depicted in Figure 15, demonstrating effectiveness in enhancing position accuracy. In Figure 15a, the scatter plot contrasts position errors at various points before and after calibration. It is clear that the position error decreases significantly, demonstrating the effectiveness of the calibration process. Figure 15b is a histogram that compares the frequency of position errors between different calibration methods. The method labeled as our method appears to achieve a higher concentration of lower magnitude errors, indicating superior performance in reducing the position error when compared to MDH, NTM, and RFCM. Figure 15c presents a 3D histogram, further emphasizing the distribution of position errors in different directions, and our method exhibits a distribution skewed towards lower errors, reinforcing its efficacy. Lastly, Figure 15d showcases a violin plot for the error distribution of each method, giving insight into the density distribution of the errors. The narrow and peaked distribution of our method suggests a tighter clustering of data points around a lower median error, while other methods display broader distributions, indicative of a wider range of error magnitudes. Overall, the data across these visuals collectively suggest that our model consistently outperforms the alternative methods in minimizing position errors.

According to Table 8, our proposed method achieved a reduction in RMS of 19.5% compared to the MDH method, and reductions of 13.8% and 9.1% compared to the NTM and RFCM, respectively. Furthermore, it exhibits optimal performance in terms of the distribution of errors and extreme values.

## 6. Discussion and Conclusions

In this paper, it is evident that the kinematic and joint compliance modeling method effectively enhances the position accuracy of robotic vision systems. Through an innovative integration of a 3D rotary laser sensor, unified kinematic modeling, joint compliance modeling, and a three-stage parameter identification process, this research offers a comprehensive solution to the challenges of system position accuracy. The two validation experiments—employing both a laser tracker and a 3D vision measurement system to assess the accuracy of the proposed models—robustly demonstrate the method’s superiority over existing models. Compared to the Modified Denavit–Hartenberg (MDH), nonlinear transmission model (NTM), and Rigid–Flexible Coupling Model (RFCM) methods, our approach proves its efficacy in enhancing the position accuracy of robotic vision system. The following discussion reviews the key aspects of the proposed model, evaluates its complexity related to previous methods, and emphasizes its potential benefits for industrial manufacturing cycles that require the highest precision.

The integration of kinematic and joint compliance factors introduces an increase in model complexity. This complexity is a direct consequence of our comprehensive approach to modeling, which accounts for factors often overlooked in simpler models, such as the elasticity of robotic joints, and the impact of external loads and links. While the proposed model demands more significant computational resources and a complex calibration process, it delivers substantial improvements in position accuracy by at least 9.1% according to the experiment results. These improvements are critical in applications where precision is paramount, outweighing the drawbacks associated with increased model complexity.

The practical implications of our model are particularly significant in high-precision industrial manufacturing cycles. The advanced accuracy offered by our modeling approach can lead to remarkable enhancements in product quality and a notable reduction in waste and rework. In industries such as industrial manufacturing, automotive assembly, and aerospace engineering, where the cost of inaccuracies can be exceptionally high, the potential savings and efficiency gains are substantial. Furthermore, the adaptability of our model to various robotic systems and its scalability across different manufacturing tasks underscore its versatility and broad applicability.

Future research will focus on streamlining the model’s complexity and enhancing its usability, striving to simplify the calibration process and reduce computational requirements without compromising the accuracy benefits. Furthermore, exploring the model’s application across a broader range of industrial scenarios will be crucial in fully realizing its potential to revolutionize precision in robotic automation.

## Figures and Tables

**Table 1:** Torque applied by the link.

Torque	Link_1_	Link_2_	Link_3_~Link_4_	Link_5_~Link_6_
$\tau_{1}$	0	0	0	—
$\tau_{2}$	—	G_2_A_2_sin(θ_2_)	G_3_L_2_sin(θ_2_) + G_3_A_3_sin(θ_2_ − θ_3_)	
$\tau_{3}$	—	—	G_3_A_3_sin(θ_2_ − θ_3_)	
$\tau_{4}$	—	—	0	
$\tau_{5}$	—	—	—	
$\tau_{6}$	—	—	—	

**Table 2:** Ceramic balls measured by CMM (mm).

Ceramic Ball	x	y	z	r
1	185.328	408.400	2303.870	14.999
2	240.396	377.943	2640.910	15.002
3	−196.154	289.749	2727.350	15.001
4	−246.181	344.340	2379.780	14.998

**Table 3:** Calibration results of TBU and TCG based on the 3D rotary laser sensor.

	Transformation	Trans (x, y, z)/mm	Rot (r, w, p)/deg
TBU	{Ceramic}→{Base} ^(User1)^	500.580, 298.611, 2638.660	−1.815, −170.153, 98.596
TCG	{Gripper}→{Cam}	7.096, 0.355, −348.800	0.825, −24.750, 179.425

**Table 4:** Calibration results based on 3D rotary laser sensor.

MDH Parameters					Joint Compliance Parameters (×10^−3^)	
Joint	θ/deg	d/mm	a/mm	α/deg	$k_{0}^{\left(1:3\right)}$	0.0690, 0.250, −0.164
1	—	—	49.780	−89.973	$k_{a1}^{\left(1:3\right)}$	−0.245, −0.261, −0.695
2	−90.066	−0.0206	330.341	179.937	$k_{a2}^{\left(1:3\right)}$	−0.177, −0.272, −0.0487
3	0.0372	0.0106	34.969	−90.001	$k_{b1}^{\left(1:3\right)}$	−0.333, −0.196, −0.610
4	0.167	−335.305	−0.0286	90.037	$k_{b2}^{\left(1:3\right)}$	−0.0437, −0.199, −0.00706
5	179.720	−0.601	−0.0766	90.103	$\lambda_{\theta }^{*}$ [mm]	diag(−1.159, 0.0640, 0.0582, 0.183, 0.852, −0.158)
6	—	—	—	—	$s_{1}^{*},s_{2}^{*}$ [mm]	319.836, 1833.495

**Table 5:** Statistical comparison based on the first experiment.

Method	Model Size	RMS (mm)	Std. (mm)	Avg (mm)	Max (mm)
MDH	30	0.0773	0.0485	0.0602	0.1947
NTM	45	0.0702	0.0428	0.0557	0.1752
RFCM	39	0.0692	0.0438	0.0537	0.1755
Ours	53	0.0574	0.0350	0.0455	0.1699

**Table 6:** Calibration results of TBU and TCG based on laser tracker.

	Transformation	Trans (x, y, z)/mm	Rot (r, w, p)/deg
TBU	{LT}→{Base} ^(User2)^	−1105.029, −955.794, −116.344	21.846, −0.521, 0.166
TCG	{Gripper}→{Reflector}	44.200, −60.458, 104.009	—

**Table 7:** Calibration results based on laser tracker.

MDH Parameters					Joint Compliance Parameters (×10^−3^)	
Joint	θ/deg	d/mm	a/mm	α/deg	$k_{0}^{\left(1:3\right)}$	0.158, 0.403, −0.0281
1	—	—	50.202	−89.979	$k_{a1}^{\left(1:3\right)}$	−0.120, −0.0430, −0.503
2	−89.876	−0.0121	330.049	179.950	$k_{a2}^{\left(1:3\right)}$	−0.0512, −0.0553, 0.144
3	−0.0293	0.2361	35.343	−89.973	$k_{b1}^{\left(1:3\right)}$	−0.206, 0.0217, −0.418
4	0.720	−335.152	−0.232	90.016	$k_{b2}^{\left(1:3\right)}$	0.0819, 0.0188, 0.183
5	−179.415	−0.0786	0.125	90.012	$\lambda_{\theta }^{*}$ [mm]	diag(−1.289, 0.0668, 0.0522, 0.138, 0.732, −0.117)
6	—	—	—	—	$s_{1}^{*},s_{2}^{*}$ [mm]	307.383, 1841.103

**Table 8:** Statistical comparison based on the second experiment.

Method	RMS (mm)	Std. (mm)	Avg (mm)	Max (mm)
MDH	0.0473	0.0282	0.0380	0.1319
NTM	0.0446	0.0266	0.0358	0.1132
RFCM	0.0424	0.0255	0.0339	0.1099
Ours	0.0381	0.0255	0.0308	0.0955

## Data Availability

Data are contained within the article.

## Nomenclature

Joints ∈ℝ	Number of robot joints
q ∈ℝ	Position of end-effector
θ ∈ℝ	Joints angle
d ∈ℝ	Link offset
a ∈ℝ	Link distance
α ∈ℝ	Twist angle
δi ∈ℝ	Error of Parameter *i*
τ ∈ℝ	Joint torque
$A_{i}\;\in \mathbb{R}$	Distance from the gravity center of link *i* to rotation axis *i*
$L_{i}\;\in \mathbb{R}$	Distance from the rotation axis *i* + 1 to rotation axis *i*
$\theta \;\in {\mathbb{R}}^{\mathrm{Joints}}$	Concatenated θ over Joints samples
$\tau \;\in {\mathbb{R}}^{\mathrm{Joints}}$	Concatenated τ over Joints samples
$\lambda \;\in {\mathbb{R}}^{\mathrm{Joints}\times \mathrm{Joints}}$	Compliant matrix
Habc ∈ℝ6×6	Force and torque expressed from frame b to c in frame a
${\mathrm{Rot}}_{i}\;\in SO(3)$	Rotation homogeneous transformation around axis i
Trans ∈SO(3)	Translation homogeneous transformation
Tab ∈SE(3)	Frame a expressed in frame b
J	Jacobian matrix
$\eta_{*}$	Parameter vector
φ	Fourier series
{⋅}	Reference frame
[⋅]	Antisymmetric matrix of vector
$\left\|\cdot \right\|$	Magnitude of vector
